# The Association of Extreme Tachycardia and Sustained Return of Spontaneous Circulation after Nontraumatic Out-of-Hospital Cardiac Arrest

**DOI:** 10.1155/2020/5285178

**Published:** 2020-06-29

**Authors:** Dong Keon Lee, Eugi Jung, You Hwan Jo, Joonghee Kim, Jae Hyuk Lee, Seung Min Park, Yu Jin Kim

**Affiliations:** ^1^Department of Emergency Medicine, Seoul National University Bundang Hospital, Seongnam, Republic of Korea; ^2^Department of Emergency Medicine, VHS Medical Center, Seoul, Republic of Korea; ^3^Department of Emergency Medicine, Seoul National University College of Medicine, Seoul, Republic of Korea

## Abstract

**Objective:**

Heart rate (HR), an essential vital sign that reflects hemodynamic stability, is influenced by myocardial oxygen demand, coronary blood flow, and myocardial performance. HR at the time of the return of spontaneous circulation (ROSC) could be influenced by the *β*1-adrenergic effect of the epinephrine administered during cardiopulmonary resuscitation (CPR), and its effect could be decreased in patients who have the failing heart. We aimed to investigate the association between HR at the time of ROSC and the outcomes of adult out-of-hospital cardiac arrest (OHCA) patients.

**Methods:**

This study was a secondary analysis of a cardiac arrest registry from a single institution from January 2008 to July 2014. The OHCA patients who achieved ROSC at the emergency department (ED) were included, and HR was retrieved from an electrocardiogram or vital sign at the time of ROSC. The patients were categorized into four groups according to the HR (bradycardia (HR < 60), normal HR (60 ≤ HR ≤ 100), tachycardia (100 < HR < 150), and extreme tachycardia (HR ≥ 150)). The primary outcome was the rate of sustained ROSC and the secondary outcomes were the rate of one-month survival and six-month good neurologic outcome.

**Results:**

A total of 330 patients were included. In the univariate logistic regression model, the rate of sustained ROSC increased by 17% as HR increased by every 10 beats per minute (bpm) (odds ratio (OR), 1.171; 95% confidence interval (CI), 1.077–1.274, *p* < 0.001). In the multivariate logistic regression model, extreme tachycardia was independently associated with a high probability of sustained ROSC compared to normal heart rate (OR, 15.96; 95% CI, 2.04–124.93, *p*=0.008).

**Conclusion:**

Extreme tachycardia (HR ≥ 150) at the time of ROSC is independently associated with a high probability of sustained ROSC in nontraumatic adult OHCA patients.

## 1. Introduction

Out-of-hospital cardiac arrest (OHCA) is still a major challenge in medicine despite many efforts to improve outcomes. In patients with OHCA, the rate of the return of spontaneous circulation (ROSC) has been reported to be from 24 to 44% [1–3]. Among patients who experience ROSC after OHCA, rearrest often develops within a short time and results in death.

Most in-hospital deaths after ROSC are due to cardiac dysfunction and neurologic failure in postcardiac arrest syndrome, and early death is mainly due to cardiac dysfunction [4–6]. Although cardiac dysfunction after ROSC is known to be reversible and cardiac function resumes after 48 to 72 hours, severe cardiac dysfunction prevents the maintenance of ROSC and may result in rearrest [7].

When ROSC is achieved, blood pressure and heart rate (HR) are usually measured immediately. HR, an essential vital sign that reflects hemodynamic stability, influences myocardial oxygen demand, coronary blood flow, and myocardial performance [8–10].

HR at the time of ROSC could be influenced by the *β*1-adrenergic effect of the epinephrine administered during cardiopulmonary resuscitation (CPR), and its effect could be decreased in patients who have the failing heart which shows *β*1-adrenergic receptor downregulation [11, 12].

In children with OHCA, it has been reported that HR in the first hour after sustained ROSC was associated with survival to discharge [13]. However, the association of HR at the time of ROSC with the outcomes of adult OHCA has yet to be investigated.

Given that the cardiac dysfunction after ROSC showed significant morbidity and mortality [14], we hypothesized that the HR at the time of ROSC, which might reflect the cardiac function, could be associated with the outcome of OHCA. Therefore, we investigated the association between HR at the time of ROSC and the probability of sustained ROSC.

## 2. Methods

### 2.1. Study Design and Setting

We performed a secondary analysis of consecutive OHCA databases in a single ED from January 2008 to July 2014. The study facility was a 1250-bed urban tertiary academic hospital with an annual ED census of approximately 85,000. This study was approved by the Institutional Review Board (IRB) of Seoul National University Bundang Hospital and reported according to the STROBE (STrengthening the Reporting of OBservational studies in Epidemiology) guidelines for reporting observational trials [15].

Patients with cardiac arrest were managed based on the recommendations of the international guidelines during the study period [16]. Prehospital CPR was provided by fire-station-based emergency medical technicians (EMTs), and the level of prehospital CPR was primarily basic life support, including chest compression/ventilation and automatic external defibrillator use.

When the prehospital EMTs notified the ED, the CPR team of the ED was activated. Details on the CPR team and resuscitation protocol in the ED have been presented previously [17]. The standard-dose epinephrine defined as 1 mg was administered intravenously every three to five minutes [16]. Blood samples were drawn during the CPR and immediately after ROSC, and they were then sent to the central laboratory for analysis. When ROSC was achieved, the vital signs were measured with noninvasive equipment (IntelliVue, MX700, Philips, Netherlands) within three minutes after ROSC and every one hour thereafter. Among them, the first measurement was used for analysis. The 12-lead electrocardiography (ECG) (Philips PageWriter TC‐70; GE, Eindhoven, the Netherlands) was also taken within three minutes after ROSC. If there was a clinical necessity, vital signs were measured and ECG was taken repeatedly.

### 2.2. Study Population

Based on the OHCA registry in accordance with Utstein-style guidelines [18], consecutive patients who were 18 years or older with nontraumatic OHCA and received advanced cardiac life support in the ED were included in this study [19, 20]. Patients under 18 years, patients who achieved prehospital ROSC or failed to achieve any ROSC, patients with traumatic cardiac arrest, patients without recorded HR due to brief ROSC of less than a minute, and patients who were rescued by extracorporeal life support (ECLS) were excluded.

Bradycardia is defined as HR below 60 beats per minute and tachycardia is defined as HR above 100 beats per minute. Given those definitions and that symptomatic tachycardia generally involves rates over 150 beats/min [21–23], the patients were categorized into four groups according to their HR at the time of ROSC as follows: bradycardia (HR < 60 beats/min), normal HR (60 ≤ HR ≤ 100 beats/min), tachycardia (100 < HR < 150 beats/min), and extreme tachycardia (HR ≥ 150 beats/min).

### 2.3. Data Collection and Outcomes

Data were collected by trained research personnel using a standardized registry template form [18]. The form included demographics, witnessed arrest, place of arrest, bystander CPR, initial rhythm, presumed cause of arrest, drugs, and the outcomes of the patient including sustained ROSC, one-month survival, and six-month neurologic outcome.

The HR of each patient was retrieved from the ECG taken at the time of ROSC. If the HR was not available from the ECG, the HR was retrieved from the vital signs recorded immediately after ROSC. *N*-terminal pro-brain type natriuretic peptide (NT-proBNP) was measured with blood samples taken immediately after ROSC in the central laboratory to investigate the association between the NT-proBNP concentration and the HR groups at the time of ROSC.

The primary outcome was the rate of sustained ROSC according to the HR groups. Sustained ROSC was defined according to the Utstein statement as the status in which chest compressions for 20 consecutive minutes are not required and signs of circulation persist [24]. The secondary outcomes were one-month survival and six-month good neurologic outcome according to the HR groups. The six-month good neurologic outcome was defined as a cerebral performance category (CPC) one or two at six months after admission, and it was investigated with structured telephone follow-up protocol performed by trained research personnel.

### 2.4. Statistical Analyses

The Chi-square test or Fisher's exact test was used for comparisons of categorical variables and presented as numbers (percentages). Continuous variables were examined with the Kolmogorov–Smirnov test for the normality of the distribution and presented as medians (interquartile ranges). The analysis of variance or the Kruskal–Wallis test was used depending on the normality of the distribution. If the Kruskal–Wallis test showed statistical significance, Bonferroni correction was used for multiple comparisons.

Univariable logistic regression analysis was performed to investigate the association between the HR (as a continuous variable) at the time of ROSC and the probability of sustained ROSC. Multivariable logistic regression analysis was performed with the enter method, which brings all clinically relevant variables into the model, to determine the independent factors for outcomes including sustained ROSC, one-month survival, and six-month neurologic outcome. As a result, HR (bradycardia, normal HR, tachycardia, and extreme tachycardia), age, sex, witnessed arrest, public place, shockable initial rhythm, bystander CPR, total epinephrine dose, mean arterial pressure at the time of ROSC, no-flow time defined as time from collapse to the start of resuscitation attempts, and low-flow time defined as time from start of life support until the restoration of spontaneous circulation were included in the multivariable logistic regression analysis.

All tests were two-sided, and *p* values <0.05 were considered to indicate statistical significance. Data were analyzed using IBM SPSS statistics version 21 (IBM, Armonk, NY, USA), and a curve fit was performed with ggplot2 software.

## 3. Results

### 3.1. Baseline Characteristics

**Figure 1:**
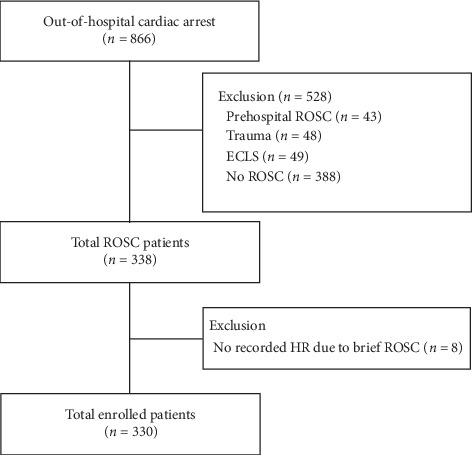
Flowchart of the study population. ROSC, return of spontaneous circulation; ECLS, extracorporeal life support; HR, heart rate; brief ROSC, ROSC less than one minute of duration.

During the study period, a total of 866 adult OHCA patients who presented to the ED were initially screened. Among these patients, 43 patients who achieved prehospital ROSC, 48 patients whose cause of cardiac arrest was trauma, 49 patients who were supported by ECLS devices, and 388 patients who did not achieve any ROSC were excluded. Among the remaining 338 patients, eight had no HR record because of brief ROSC (duration less than one minute). As a result, 330 OHCA patients who underwent CPR in the ED and had HR records after ROSC were included in the final analysis (Figure 1).

The median age of the patients was 72 (59–79) years, and 194 (58.8%) patients were males. HR was retrieved from the ECGs taken immediately after ROSC in 323 patients and from the vital signs in seven patients. The numbers of patients according to HR were 26 in the bradycardia group, 67 in the normal HR group, 176 in the tachycardia group, and 61 in the extreme tachycardia group. The mean arterial pressure at the time of ROSC was higher in the extreme tachycardia group than in the other groups. The total epinephrine dose was lower in the extreme tachycardia group compared to the other groups. The median low-flow time showed no significant difference between groups after Bonferroni correction. There was no significant difference among groups in terms of the age, gender, rates of witnessed arrest, public place, initial shockable rhythm, bystander CPR, and no-flow time (Table 1).

### 3.2. Resuscitation Outcomes according to the HR Groups

**Figure 2:**
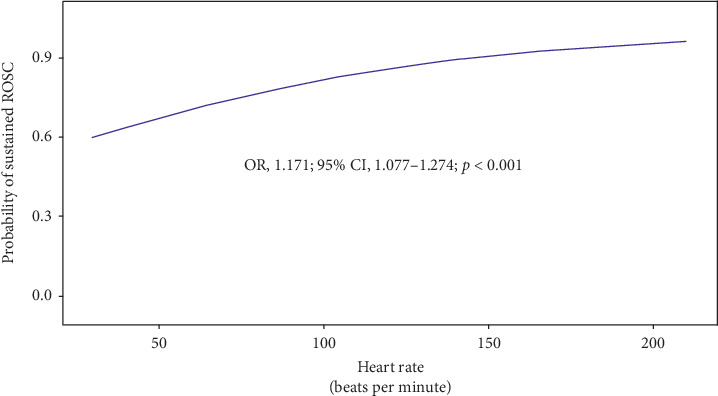
The probability of sustained ROSC according to heart rate. ROSC, return of spontaneous circulation.

In terms of the outcomes, the sustained ROSC was achieved in 60 of 61 patients (98.4%) in the extreme tachycardia group, and the rate of the sustained ROSC was highest in the extreme tachycardia group and lowest in the bradycardia group (Table 1). In the univariable logistic regression model, the probability of sustained ROSC increased by 17% as HR increased by every 10 beats per minute (OR, 1.171, 95% confidence interval [CI], 1.077–1.274, *p* < 0.001) (Figure 2). The rates of one-month survival and six-month good neurologic outcome were not different between the four groups (Table 1).

In the multivariable logistic regression analysis, the extreme tachycardia group was independently associated with a high probability of sustained ROSC compared to the normal HR group (OR 11.297; 95% CI 1.350–94.527; *p*=0.025, Table 2).

### 3.3. NT-proBNP Concentration and the HR Groups

**Figure 3:**
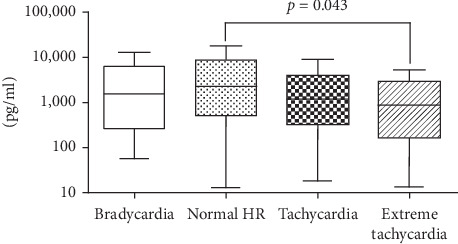
*N*-terminal pro-brain-type natriuretic peptide concentration. Bradycardia, heart rate (HR) < 60 beats/min; normal, 60 ≤ HR ≤ 100 beats/min; tachycardia, 100 < HR < 150 beats/min; extreme tachycardia, HR ≥ 150 beats/min.

The median NT-proBNP concentration in total patients was 1403 [277–4579] pg/mL. The NT-proBNP concentration was significantly lower in the extreme tachycardia group than in the normal HR group (874 [164–2902] pg/mL and 2277 [519–8743] pg/ml in the extreme tachycardia group and normal HR group, respectively; *p*=0.043; Figure 3). There was no difference between the bradycardia, normal HR, and tachycardia groups.

## 4. Discussion

In the present study, patients with extreme tachycardia (HR ≥ 150 beats/min) had the highest rate of sustained ROSC, and extreme tachycardia was an independent factor for the probability of sustained ROSC.

The myocardium is damaged by ischemia and reperfusion injury and electrical shock during cardiac arrest and resuscitation. It leads to reduced contractility and compliance of myocardium, and severe myocardial dysfunction has been reported to be associated with high mortality in the early postcardiac arrest period [25, 26].

Myocardial dysfunction after cardiac arrest has been measured directly or indirectly using pulmonary artery catheterization, echocardiography, blood pressure, and several biomarkers such as troponin and NT-proBNP concentrations. Low cardiac output measured by pulmonary artery catheterization was common in patients after ROSC from cardiac arrest, but the association of cardiac output with outcomes was unclear [27, 28]. Echocardiography has been widely used to measure the myocardial function after ROSC. The systolic and diastolic dysfunctions were observed after ROSC, and they were associated with outcomes [7, 26, 29]. Hypotension and vasopressor support after ROSC were associated with ischemic insult leading to cardiovascular failure, which could predict worse outcomes after cardiac arrest [30–32]. Troponin and NT-proBNP have also been investigated in patients with cardiac arrest although the results were reported differently at the time of measurement [33].

In this study, extreme tachycardia was associated with the high probability of sustained ROSC. However, Redwood et al. reported that higher HR in acute myocardial ischemia was associated with increased myocardial infarct size in the experimental model, while reduced HR may show a beneficial effect because of the decrease in myocardial oxygen consumption and prolongation of total diastolic time which affects coronary collateral flow [34].

This difference could be explained by the *β*1-adrenergic receptor downregulation. Epinephrine is an *α*- and *β*-adrenoceptor agonist and was administered every three minutes during CPR [35]. The *α*-adrenergic effect increases diastolic aortic pressure and coronary and cerebral perfusion pressure, whereas the *β*1-adrenergic effect increases HR and contractility [21, 29]. Given that *β*1-adrenergic receptor downregulation occurs in postcardiac arrest myocardial dysfunction and failing heart [11, 12], extreme tachycardia could be the response of epinephrine in the better-conditioned heart. This is supported by the lowest NT-proBNP concentration of the extreme tachycardia group. Hence, the patients who showed extreme tachycardia at the time of ROSC might have better-conditioned hearts that show preserved *β*1-adrenergic receptors and concomitant better responses to epinephrine than the others.

Although extreme tachycardia showed a high rate of sustained ROSC, it showed no association with the six-month good neurologic outcome. Nevertheless, HR is still important since early prognostication is valuable to the determination of the treatment and disposition plan for OHCA patients, and it is an easily and immediately measurable parameter. Based on the results of the present study, patients with extreme tachycardia after ROSC should be paid more attention in regard to postresuscitation care such as primary coronary intervention and targeted temperature management, while hemodynamic support should be continued for all patients including those without extreme tachycardia after ROSC.

This study has some limitations. First, this study was performed retrospectively in a single institution. Therefore, the results might not be generalizable to other institutions, so a multicenter study is warranted.

Second, echocardiography was not performed immediately after ROSC, so the exact cardiac function could not be investigated. Instead, NT-proBNP was used as an indicator of cardiac function.

Third, we analyzed HR only at the time of ROSC and did not analyze the change with time. Depending on cardiac function and type and dose of vasopressors and inotropes, HR could be different with time. However, although we mainly retrieved HR from the ECG taken immediately after ROSC, HR could be easily measured with cardiac monitoring. Therefore, it is worth noting that a simple HR measurement could be used as an independent predictor of sustained ROSC.

## 5. Conclusions

Extreme tachycardia (HR ≥ 150) at the time of ROSC is associated with a high probability of sustained ROSC in nontraumatic adult OHCA patients.

## Figures and Tables

**Table 1:** Baseline characteristics and the outcomes of the patients.

	Total	Bradycardia	Normal HR	Tachycardia	Extreme tachycardia	*p*
	(*n* = 330)	(*n* = 26)	(*n* = 67)	(*n* = 176)	(*n* = 61)	
Age (years)	72 (59–79)	73 (66–77)	74 (62–79)	72 (60–79)	69 (49–76)	0.094
Male	194 (58.8)	17 (65.4)	44 (65.7)	98 (55.7)	35 (57.4)	0.470
Witnessed arrest	264 (80.0)	20 (76.9)	57 (85.1)	141 (80.1)	46 (75.4)	0.565
Public place	52 (15.8)	5 (19.2)	11 (16.4)	23 (13.1)	13 (21.3)	0.451
Shockable initial rhythm	41 (12.4)	3 (11.5)	7 (10.4)	21 (11.9)	10 (16.4)	0.757
Bystander CPR	105 (31.8)	10 (38.5)	18 (26.9)	55 (31.3)	22 (36.1)	0.611
MAP at ROSC (mmHg)	93 (68–114)	81 (43–94)	89 (67–105)	89 (68–113)	111 (85–137)	<0.001^*∗*^
HR at ROSC (beats/min)	120 (98–144)	47 (32–51)	84 (76–94)	124 (113–136)	161 (155–175)	<0.001^*∗*^
No-flow time (min)	4 (0–12)	4 (1–14)	5 (0–12)	4 (0–11)	1 (0–9)	0.194
Low-flow time (min)	26 (17–36)	37 (18–46)	27 (18–36)	25 (17–34)	24 (15–32)	0.047^†^
Total epinephrine dose (mg)	3 (2–4)	3 (2–7)	4 (2–5)	3 (2–4)	2 (1–3)	<0.001^*∗*^
Sustained ROSC	281 (85.2)	18 (69.2)	54 (80.6)	149 (84.7)	60 (98.4)	0.002^‡^
One-month survival	58 (17.8)	4 (15.4)	11 (16.1)	26 (15.1)	17 (27.9)	0.153
Good neurologic outcome	23 (7.0)	2 (7.7)	4 (6.0)	5 (6.0)	5 (6.0)	0.965

Data are expressed as median (interquartile range) or number (%) as appropriate. Bradycardia, HR < 60 beats/min; normal, 60 ≤ HR ≤ 100 beats/min; tachycardia, 100 < HR < 150 beats/min; extreme tachycardia, HR ≥ 150 beats/min; HR, heart rate; CPR, cardiopulmonary resuscitation; MAP, mean arterial pressure; ROSC, return of spontaneous circulation. ^*∗*^Statistically significant after Bonferroni correction. ^†^Statistically not significant after Bonferroni correction. ^‡^Statistically significant.

**Table 2:** Multivariable logistic regression analysis for the outcomes.

	Sustained ROSC			One-month survival				Six-month good neurologic outcome			
	OR	(95% CI)	*p*		OR	(95% CI)	*p*		OR	(95% CI)	*p*
Heart rate group											
Bradycardia	0.79	(0.23–2.75)	0.711		1.92	(0.41–9.10)	0.411		1.69	(0.29–9.82)	0.558
Normal HR	1.00	(Reference)			1.00	(Reference)			1.00	(Reference)	
Tachycardia	1.23	(0.54–2.82)	0.624		0.70	(0.27–1.79)	0.449		0.79	(0.29–2.16)	0.642
Extreme tachycardia	11.30	(1.35–94.53)	0.025^∗^		0.82	(0.27–2.49)	0.724		0.92	(0.28–3.08)	0.896
Age, per 10 years	0.85	(0.66–1.11)	0.230		0.82	(0.66–1.02)	0.079		0.80	(0.63–1.02)	0.073
Male	0.73	(0.34–1.57)	0.427		2.15	(1.02–4.55)	0.045^∗^		3.45	(1.56–21.50)	0.009^*∗*^
Witnessed arrest	2.15	(0.91–5.11)	0.083		1.30	(0.49–3.46)	0.605		0.96	(0.34–2.65)	0.929
Public place	1.84	(0.56–6.02)	0.313		1.11	(0.42–2.96)	0.829		1.83	(0.66–5.07)	0.245
Shockable initial rhythm	1.19	(0.37–3.84)	0.268		3.03	(1.22–7.51)	0.017^∗^		3.18	(1.25–8.10)	0.015^*∗*^
Bystander CPR	1.67	(0.67–4.16)	0.268		1.81	(0.76–4.34)	0.182		1.87	(0.71–4.91)	0.205
Total epinephrine dose, per 1 mg	0.89	(0.75–1.05)	0.152		0.69	(0.52–0.92)	0.011		0.77	(0.57–1.04)	0.083
MAP ≥ 80 mmHg at ROSC	0.90	(0.43–1.88)	0.778		2.21	(0.98–4.97)	0.056		1.84	(0.76–4.43)	0.175
No-flow time, per 1 min	1.02	(0.98–1.06)	0.395		0.95	(0.91–1.00)	0.057		0.94	(0.89–0.99)	0.042^∗^
Low-flow time, per 1 min	0.99	(0.97–1.02)	0.716		0.95	(0.91–0.98)	0.005^∗^		0.94	(0.91–0.98)	0.007^∗^

Bradycardia, HR < 60 beats/min; normal, 60 ≤ HR ≤ 100 beats/min; tachycardia, 100 < HR < 150 beats/min; extreme tachycardia, HR ≥ 150 beats/min. ROSC, return of spontaneous circulation; OR, odds ratio; CI, confidence interval; HR, heart rate; CPR, cardiopulmonary resuscitation; MAP, mean arterial pressure. ^*∗*^Statistically significant.

## Data Availability

The data used to support the findings of this study have not been made available because the authors were not allowed to share the patients' information.
